# Emerging trends in invasive and noninvasive isolates of *Streptococcus agalactiae*in a Latin American hospital: a 17-year study

**DOI:** 10.1186/1471-2334-14-428

**Published:** 2014-08-03

**Authors:** Maria del Pilar Crespo-Ortiz, Claudia Rocio Castañeda-Ramirez, Monica Recalde-Bolaños, Juan Diego Vélez-Londoño

**Affiliations:** Department of Biomedical Sciences, Santiago de Cali University, Cali, Colombia; Department of Microbiology, University of Valle, Cali, Colombia; Microbiology and Infectious Diseases Division, Foundation Valle del Lili Hospital, Cali, Colombia; Bacteriology School, University of Valle, Cali, Colombia

**Keywords:** Neonatal infections, Immunosuppression, Bacteremia, *Streptococcus agalactiae*, Group B *Streptococcus*

## Abstract

**Background:**

*Streptococcus agalactiae* or group B Streptococcus (GBS) has been recognized as a lethal pathogen in neonates worldwide. *S. agalactiae* infections also severely affect pregnant women and immunosuppressed adults with substantial attributable morbidity and mortality. However, in Latin America, studies on the epidemiology and behaviour of *S. agalactiae* infections remain limited.

**Methods:**

To better understand the behaviour of *S. agalactiae* infections in our region, we conducted a retrospective study to phenotypically describe *S. agalactiae* isolates collected in one of the largest hospitals in Colombia at two time periods: 1994–2001 and 2004–2012. The isolates were identified by biochemical analysis and tested for antimicrobial susceptibility.

**Results:**

In 1994–2001 a total of 201 *S. agalactiae* isolates were found in urine 38.3%, vaginal exudates 27.8%, soft tissue 12.9%, and blood 8.5%. Susceptibility to ampicillin or penicillin was 94% whereas resistance to erythromycin and clindamycin were 2.8% and 5.2% respectively. In total 46 culture-positive cases of invasive infections were reported, 11 (24%) in neonates and 35 (76%) in adults. In 2004–2012 a total of 671 isolates were found in urine 47.8%, vaginal exudates 32.6%, soft tissue 2.7% and blood 9%. Susceptibility rates to ampicillin and penicillin were 98% whereas resistance to erythromycin and clindamycin were 12.5% and 9.4%. A total of 95 severe infections were reported: 12 (12.6%) were in neonates, 5 (5.3%) in children and 78 (82.1%) in adults. Over the 17-year study period the averaged prevalence of invasive *S. agalactiae* isolates was 17.4%. The estimated incidence for neonatal infections was 1.34 per 1000 livebirths (0.99 × 1000 livebirths for early- onset disease and 0.35 × 1000 livebirths for late- onset disease) whereas for non-pregnant adults the estimated incidence was 0.75 × 1000 admissions.

**Conclusions:**

A remarkable increase in bloodstream infections in immunosuppressed adults and a shift to early neonatal *S. agalactiae* infections were seen over time. We also found an increase in *S. agalactiae* resistance to erythromycin and clindamycin during the study period, and the emergence of penicillin-nonsusceptible isolates. Our findings are consistent with the global trends described elsewhere, reinforcing the need for *S. agalactiae* control measures in our region.

**Electronic supplementary material:**

The online version of this article (doi:10.1186/1471-2334-14-428) contains supplementary material, which is available to authorized users.

## Background

*Streptococcus agalactiae* (Group B Streptococcus, GBS) is a colonizing bacterium in the gastrointestinal and genitourinary tracts of healthy adults, particularly in women [1–3]. Since 1970, S*. agalactiae* has been considered a very significant cause of severe neonatal infections with high morbidity and mortality. It also affects pregnant women, non-pregnant adults with underlying conditions and the elderly [4]. Maternal *S. agalactiae* colonization has been associated with early-onset neonatal septicemia (<7 days of age) and meningitis with mortality rates up to 60% [5]. The highest rates of early neonatal infection occur in premature and low-birth-weight infants due to their immature immune systems. By contrast, late-onset infections (>7 days of age) have been associated with *S. agalactiae* virulence, although less lethal, these infections are considered nosocomial and may lead to neurologic sequels in 30% of survivors [6, 7]. After 1990, in developed countries *S. agalactiae* screening and risk-based intrapartum chemoprophylaxis significantly reduced mortality to 5% [8]. However twenty years later, regardless a change in the epidemiology, *S. agalactiae* is still an important cause of severe infections. Intrapartum chemoprophylaxis has reduced the early-onset disease in neonates [9, 10] whereas the late-onset disease remains stable [10]. Recently, a remarkable increase in *S. agalactiae* invasive infections in immunocompromised adults and the elderly has been observed. Worryingly, the estimated mortality attributable to *S. agalactiae* severe infections in the elderly is more than 50% [11, 12]. Another raising concern is the potential emergence of tolerance to penicillin [13], the drug of choice for prophylaxis and therapy of *S. agalactiae* infections, and the resistance to clindamycin and erythromycin which are commonly used in patients with a history of beta-lactams allergy [8].

Although the highest burden of *S. agalactiae* infections has been reported in industrialized countries, *S. agalactiae* maternal colonization and invasive infections have been also seen in non-industrialized nations [14–17]. Nevertheless, studies on the characterization and epidemiology of *S. agalactiae* are still limited in developing countries and implementation of control strategies remains undefined due to the lack of supporting studies [18]. Severe neonatal infections and fatal cases caused by *S. agalactiae* have been reported in Latin America [7], however the behaviour and trends of *S. agalactiae* in non-pregnant adults are unknown and somehow underestimated [7]. To better understand the behaviour of *S. agalactiae* infections in our region, we have described the epidemiological, clinical and microbiological characteristics of invasive and noninvasive *S. agalactiae* isolates from patients admitted to a tertiary care hospital in Colombia over a 17-year period. We hypothesize that emerging trends in *S. agalactiae* epidemiology are similar to those from high income countries and continuous surveillance and control measures should be adopted.

## Methods

### Patients

This retrospective and cross-sectional study included clinical and microbiological records from patients with *S. agalactiae* isolates admitted in a tertiary care hospital at two time periods. Our hospital is a university-affiliated institution serving a population of approximately 700,000 located in Cali (western Colombia), the third most populous city in the country. This national reference hospital has capacity for 500 beds, 180 of them in intensive care unit (ICU), it also has specialized units for transplantation, cancer surgery and dialysis. The medical center and the laboratory also offer a wide range of outpatient services and community-level healthcare.

A first survey was conducted from 1994 to 2001. Information of *S. agalactiae* isolates was collected from the laboratory records (MicroScan, Baxter Scientific software). Invasive and noninvasive isolates were identified and clinical data from invasive infections were captured from the hospital’s patient files. Following the implementation of a new laboratory system, a second study period started from 2004 to 2012 and microbiology and clinical data were obtained as for the first survey (MicroScan LabPro software program; WalkAway Baxter Scientific). During the transition between laboratory systems (2002–2003) microbiology data were not available.

### Isolates

*S. agalactiae* isolates were identified by conventional methods based on hemolysis, colony morphology, catalase reaction and biochemical tests (Microscan WalkAway, Baxter Scientific), in some cases (<10%) bacterial identification was confirmed using the Vitek 2XL, bioMérieux system. Antimicrobial susceptibility testing was routinely performed using standard broth microdilution (Microscan WalkAway, Baxter Scientific). The minimum inhibitory concentration (MIC) was determined for penicillin, ampicillin, clindamycin, erythromycin, tetracycline and vancomycin. From 2004 to 2012, linezolid and levofloxacin were also included for susceptibility testing. Interpretive standards published by the Clinical and Laboratory Standards Institute (CLSI) were used to categorize the MICs as susceptible, intermediate, or resistant [19]. Microbiology laboratory was enrolled in periodical internal and external quality control programs to assure accuracy.

An invasive isolate was defined as that obtained from usually sterile body sites including blood, soft tissue (abscesses, ulcers and wounds) cerebrospinal fluid and synovial, peritoneal or pleural specimens. Isolates from urine, sputum and secretions were considered noninvasive. This work was approved by the Ethics Committee of the Foundation Valle del Lili Hospital (Number 170–2012).

### Statistical analysis

Demographic and clinical data from each patient with severe infection were obtained from clinical records in a data collection form. Live birth and adults admission figures were provided by the hospital statistics department. Univariate and multivariate analysis were conducted using Epi info version 7 (CDC). For comparison of clinical and epidemiological variables Chi square and Fisher’s exact test were used as appropriate. A p value <0.05 was considered significant.

## Results

From 1994 to 2001 a total of 201 *S. agalactiae* isolates were identified, 139 (69.2%) were from outpatient samples and 62 (30.8%) from inpatients samples. There were 152 women and 49 men (ratio 3.1:1). Isolates were recovered from urine 38.3%, vaginal exudates 27.8%, soft tissue 12.9%, blood 8.5%, genital male secretions 6%, sterile fluids 2% and other sources 4.5%.

**Figure 1:**
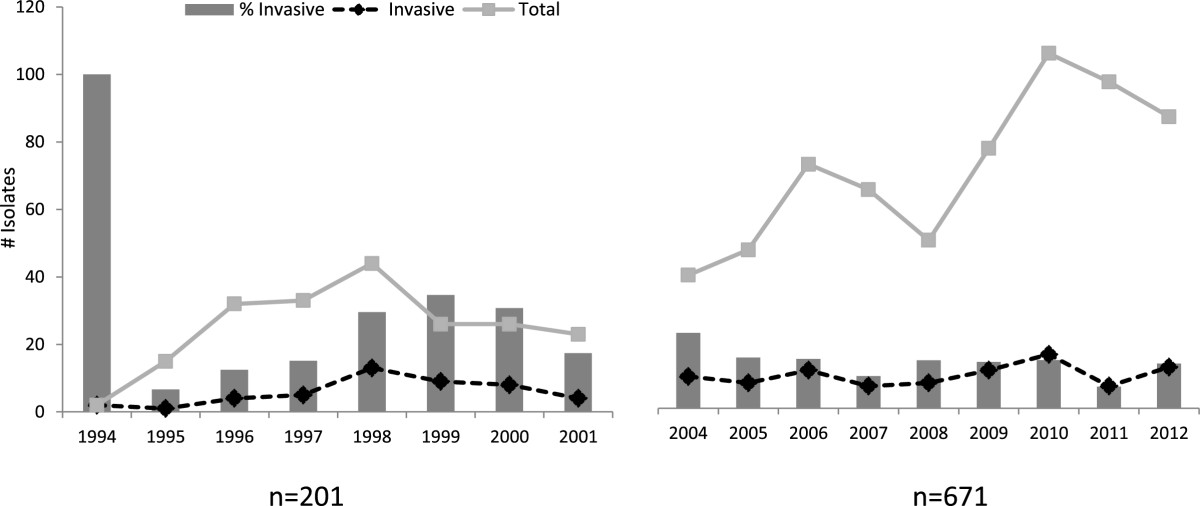
**Annual distribution of** ***S. agalactiae*** **isolates over the study period.**

The annual frequency of *S. agalactiae* is shown in Figure 1. An increase in the total of isolates was observed between 1996 and 1998, but no particular reasons for this were identified. In this 8-year period, 22.9% were considered *S. agalactiae* invasive isolates ranging from 6 to 34%, higher prevalence rates were observed between 1998 and 2000 with 65% of the total cases occurring in this period.

Six hundred and seventy one isolates were recovered during the last 9-year period (2004 to 2012), 530 (79%) from outpatient samples and 141 (21%) were from hospitalized patients. From the total of 671 patients, 540 were females and 131 were males (ratio 4.1:1). Sites of isolation included: urine 47.8%, vaginal exudates 32.6%, soft tissue 2.7%, blood 9%, sterile fluids 2.5% genital male secretions 1.3% and other sources 4.1%. There were considerably more *S. agalactiae* isolates in the recent years; the number of isolates increased from 82 in 2009 to 112 in 2010 (Figure 1). From 2004 to 2012, 14% *S. agalactiae* isolates were classified as invasive isolates ranging from 6.8% to 23.8%.

Incidence for neonatal infections for the 17-year period was 1.34 per 1000 livebirths (95% CI: 0.85-2.0). The incidence rate for early- onset infections was 0.99 per 1000 livebirths (95% CI 0.57-1.5 per 1000) and the late- onset incidence was 0.35 per 1000 livebirths (95% CI 0.16- 0.84). For non-pregnant adults, overall incidence was 0.75 severe cases per 1000 admissions (95% CI: 0.61- 0.89).

### Invasive *S. agalactiae*infections

**Figure 2:**
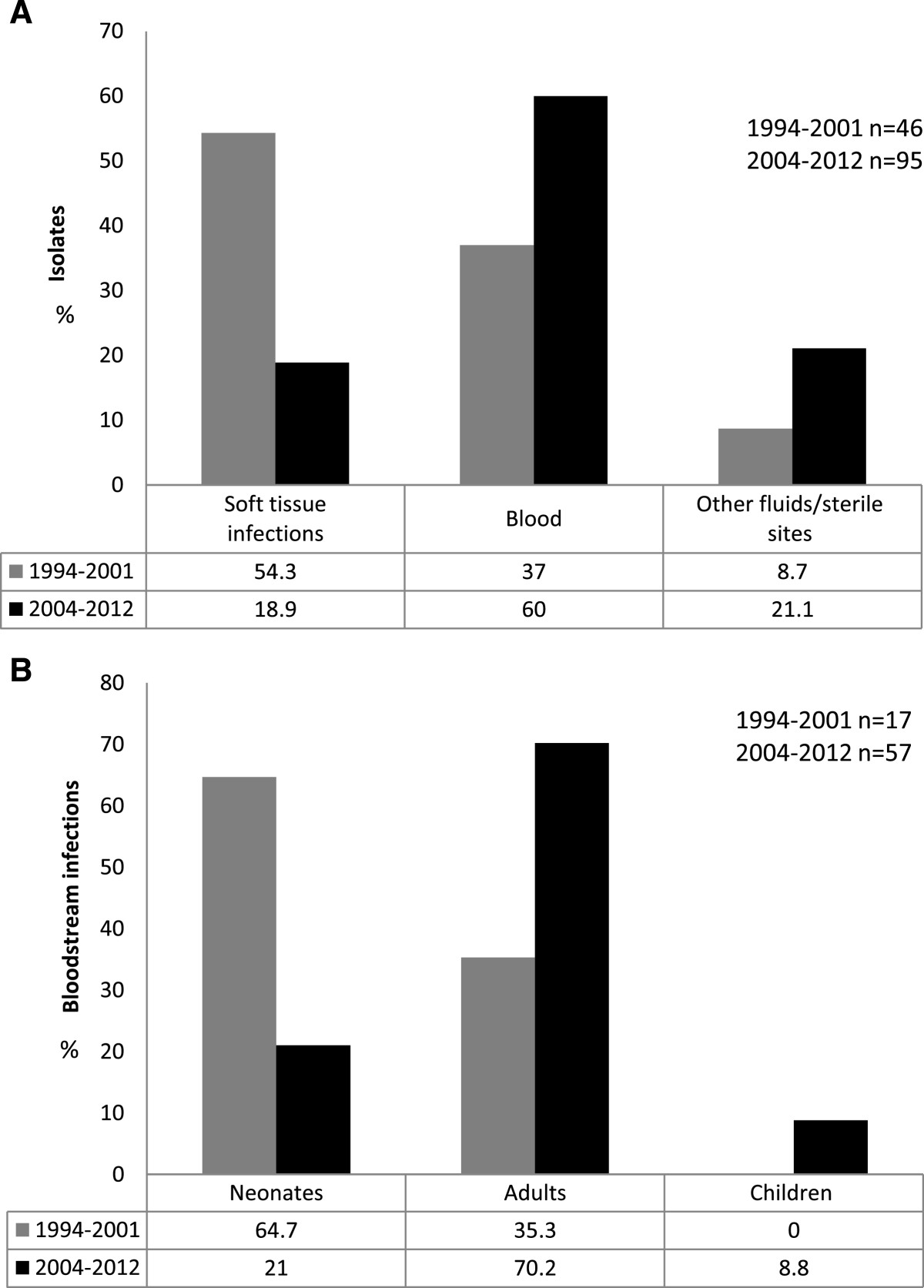
***S. agalactiae*** **invasive isolates in the study population. A)** *S. agalactiae* invasive infections distributed by site of isolation, 1994–2001 and 2004–2012. **B)** Distribution of *S. agalactiae* bloodstream infections, 1994–2001 and 2004–2012.

Noninvasive isolates were mainly recovered from outpatient samples (84%) and invasive isolates obtained from inpatient samples (63%). From 1994 to 2001 a total of 46 cases of severe infections were reported, 11 (24%) in neonates and 35 (76%) in adults. Invasive isolates were recovered from soft tissue 25 (54.3%), blood 17 (37%) and sterile fluids 4 (8.7%) (Figure 2A). Seventeen cases of bacteremia were observed: 11 (64.7%) were in neonates (6 early- and 5 late-onset infections) and 6 (35.3%) in adults (Figure 2B). The estimated incidence for *S. agalactiae* neonatal infections in this period was 1.7 per 1000 livebirths (95% CI 0.86-3.0). The incidence rates for early and late-onset disease were 0.9 per 1000 livebirths (95% CI 0.35-2.0) and 0.79 × 1000 livebirths (95% CI 0.26- 1.8) respectively. Overall mortality for neonates was 36.4% (4/11), all deaths occurred in patients with early-onset infections.

Soft tissue infections prevailed in non-pregnant adults (71.4%, 25/35) only 28.6% (10/35) of the invasive isolates were recovered from blood or sterile fluids. No in-hospital deaths were reported in adults. The estimated incidence for invasive infections in non-pregnant adults was 0.79 per 1000 admissions (95% CI 0.55-1.1).

For the second period *S. agalactiae* was recovered from 95 cases of severe infections, 12 (12.6%) neonates, 5 (5.3%) children and 78 (82%) adults. Invasive isolates were obtained from blood 57 (60%), soft tissue 18 (18.9%), sterile fluids 17 (17.9%) and 3 (3.2%) catheters (Figure 2A). Fifty seven patients with bacteremia were reported: 12 (21%) were in neonates (11 early- and one late-onset infection), 5 (8.8%) in children and 40 (70.2%) in adults (Figure 2B). Mortality rate for neonates was lower than that for the first period (16.6%, 2/12), two neonates died; one with an early-onset infection and one with late-onset infection. Risk factors identified in early neonatal infections were chorioamnionitis (3 cases), gestational diabetes (1 case), prematurity (4 cases), hemolytic disease of the newborn ABO (1 case) and maternal fever (1 case). The only late onset infection was considered nosocomial. In this study period, the estimated incidence for *S. agalactiae* neonatal disease was 1.1 per 1000 live births (95% CI 0.58-1.9), mostly early-onset infections (1 per 1000 livebirths), and the incidence for late- onset disease was 0.09 per 1000 livebirths (CI 95%: 0.002-0.5).

*S. agalactiae* invasive infections affected five children ranging in age from 5 months to 7 seven years. All infants had underlying conditions including hepatic disorders (3/5), cancer (1/5) and major surgical procedures (1/5).

The estimated incidence for invasive infections in adults was 0.73 per 1000 admissions (95% CI 0.57-0.90). Most patients with invasive *S. agalactiae* infections (91%, 52/57) had at least one chronic underlying condition such as: metastatic cancer, diabetes or transplant history. Twenty of the 57 patients (35%) had more than one underlying condition or predisposing factor (Table 1). No predisposing conditions were clearly identified in 5 cases: it was assumed that in two patients *S. agalactiae* may spread from the gastrointestinal tract to blood, one patient had an abdominal trauma before *S. agalactiae* infection and one patient had acute self-limited gastroenteritis before having *S. agalactiae* bacteremia. The remaining 3 patients developed *S. agalactiae* bone infections, one patient had septic arthritis and other patient suffered spondylodiscitis with no other associated risk factor or condition. The third patient with bone infection had morbid obesity. In none of the three patients could the primary site of dissemination be identified, *S. agalactiae* was not found in other infection sites despite being isolated from blood.

**Table 1:** **Clinical characteristics of** ***S. agalactiae*** **invasive infections in non-pregnant adults 2004 – 2012**

	Total Invasive infections n = 57 n (%)	Female n = 21 n (%)	Male n = 36 n (%)	Bloodstream Infections n = 34 n (%)
Female	21 (36.8)			12 (35.3)
Male	36 (63.2)			22 (64.7)
≥60 y.o	25 (43.9)	8 (38)	15 (42)	18 (53)
Mean		53 y. o.	55 y. o.	
Range		[17–82]	[24–83]	
In-hospital fatality	10 (17.5)	3 (14.3)	6 (16.7)	7 (20.6)
Polymicrobial infections	13 (22.8)	2 (9.5)	11 (31)	7 (20.6)
Bloodstream infections	34 (59.7)	12 (57.1)	22 (61.1)	
**Underlying conditions**				
Cancer	14 (24.6)	8 (38)	6 (16.7)	10 (29.4)
-Hematologic malignancies	7 (12.3)	4 (19)	3 (8.3)	5 (14.7)
Diabetes	16 (28)	6 (28.6)	10 (27.8)	7 (20.6)
Transplant	4 (7)	-	2 (5.6)	1 (2.9)
Cirrhosis/liver disorder	4 (7)	1 (4.8)	3 (8.3)	3 (8.8)
Renal disorder	7 (12.3)	1 (4.8)	2 (5.6)	3 (8.8)
Lung disease	3 (5.3)	2 (9.5)	2 (5.6)	2 (5.9)
HIV	3 (5.3)	-	3 (8.3)	1 (2.9)
Autoimmune disorder	3 (5.3)	1 (4.8)	1 (2.8)	3 (8.8)
Other	3 (5.3)	2 (9.5)	2 (5.6)*	2 (5.9)**
More than one underlying condition	20 (35)	6 (28.6)	14 (38.9)	13 (38.2)
**Portal of entry**				
Primary bacteremia				14 (41.2)
Skin and soft tissue				8 (23.5)
Respiratory tract				7 (20.6)
Gastrointestinal tract				4 (11.8)
Urinary tract				1 (2.9)

y.o: years old.

*No underlying condition was identified in 5 cases.

**No underlying condition was identified in 2 bloodstream infections.

*S. agalactiae* invasive infections had a similar behaviour in male and female patients. However, females had two times higher percentage of cancer (38%) than male (16.7%) by contrast liver disorders were more frequently seen in male (4.8% women vs 8.3% men). Polymicrobial infections were also more frequent in male (Table 1). In non-pregnant adults, 34 bloodstream infections were documented. Bacteremia was more prevalent in male and the elderly particularly in those aged 60 or over, clinical features of these cases are described in Table 1. Interestingly, 7 (20.6%) patients had mixed bloodstream infections. Other clinical syndromes associated with *S. agalactiae* infections were abscesses and cellulitis 8.2%, arthritis 5.5% and peritonitis 5.5%. In 13 cases (22.8%) mixed infections were reported.

A total of 40 (70%) patients with invasive isolates were admitted in ICU with complications mainly associated to neurological disorders and respiratory distress. Overall in-hospital mortality in non-pregnant adults was 19.2% and 24.1% in patients with bloodstream infections, however, neither age nor bloodstream invasion of *S. agalactiae* were significantly associated with fatality (p = 0.4 and p =0.34 respectively).

### Noninvasive infections

Over the study period a great number of isolates were obtained from urine samples. A total of 398 isolates from urine were recovered from 322 (81%) female and 76 (19%) male patients with a ratio 4.2:1. Semi-quantitative bacteriuria was determined in 161 patients. The mean of the counts was 75.750 Â± 27.507 CFU/ml, with no statistical difference between men and women (74.661 Â± 28.245 CFU/ml and 81.111 Â± 23.260 CFU/ml respectively). However 54% of the bacteriuria counts were <100.000 CFU/ml and 46% were ≥100.000 CFU/ml. Only 3.1% of the infections were polymicrobial and 96.9% were single-microorganism infections.

### Susceptibility

In 1994–2001 susceptibility rates to penicillin and ampicillin were 94% (189/201) with MIC breakpoints â‰¤0.12 and â‰¤ 0.25 μg/ml respectively 6% (12/201) of *S. agalactiae* isolates had a MIC in the 0.25 to 2 μg/ml range which is considered at the upper range of expected susceptibility [20]. Susceptibility rates to other antimicrobials were: 97.2% erythromycin, 94.8% clindamycin, 21.4% tetracycline and 100% vancomycin.

From 2004 to 2012 susceptibility rates for both penicillin and ampicillin were 98.6%. Higher MICs to penicillin (≥0.25 μg/ml) or ampicillin (≥0.5 μg/ml) were observed in 1.4% isolates Eight isolates showed MICs ≥ 2 μg/ml for ampicillin, 3 of them were recovered from sterile sites and the remaining five from genital secretions. Six isolates exhibited decreased susceptibility to penicillin; two were recovered from sterile sites. Penicillin nonsusceptible isolates showed MICs ≥2 μg/ml, three of them with reduced susceptibility to ampicillin and the other three were resistant to erythromycin or tetracycline. In 2012, we found two *S. agalactiae* isolates showing a substantially reduced susceptibility to penicillin with MICs >8 μg/ml, one of them obtained from catheter, also showed reduced susceptibility to ampicillin (MIC 4 μg/ml) and resistance to erythromycin (MIC >4 μg/ml). The remaining isolate recovered from urine showed decreased susceptibility to linezolid (MIC 2 μg/ml). Unfortunately these strains were not saved for further testing.

**Figure 3:**
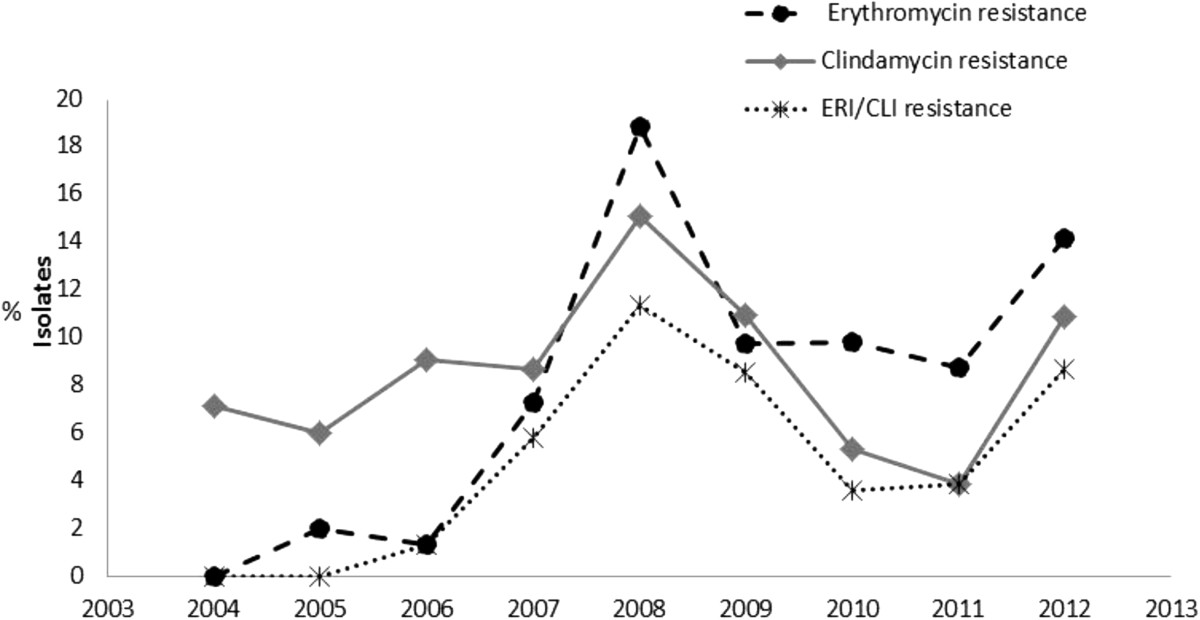
**Erythromycin and clindamycin resistance in** ***S. agalactiae*** **isolates, 2004-2012.** Erythromycin (ERI) resistance (dashed line) and clindamycin (CLI) resistance (grey solid line) are shown by year of isolation. Concurrent resistance to both clindamycin and erythromycin is also shown (dotted line with asterisk).

Susceptibility rates to erythromycin and clindamycin were 87.5% and 90.6% respectively. A significant increase in erythromycin resistance was observed over the two study periods (2.8% in 1994–2001 vs 12.5% in 2004–2012 p = 0.0001). A monitoring of the resistance over 2004 to 2012 revealed an increase in erythromycin resistance since 2007 with the highest prevalence rates in 2008 and 2012 (Figure 3). Among the total isolates, 8.9% (60/671) were only resistant to erythromycin, 2.4% (16/671) to clindamycin and concurrent resistance to both clindamycin and erythromycin was identified in 3.7% (25/671). 24.8% of the erythromycin resistant isolates showed constitutive resistance to macrolide-lincosamine-streptogramine (MLS). Susceptibility to other antibiotics was: 97.5% levofloxacin, 100% linezolid, 11.6% tetracycline and 99.7% vancomycin. Additionally 98.8% isolates had MICs <1 μg/ml for daptomycin and rifampicin. No differences were observed in the susceptibility profile of invasive and noninvasive *S. agalactiae* only a slight decrease in the susceptibility MICs to ampicillin was observed in invasive isolates (p = 0.02) (Table 2).

**Table 2:** **Antimicrobial susceptibility profiles for invasive and noninvasive** ***S agalactiae*** **isolates**

Antibiotic	1994-2001***S. agalactiae***susceptibility (%)		2004-2012***S. agalactiae***susceptibility (%)	
	Invasive	Noninvasive	Invasive	Noninvasive
Ampicillin	100	98.3	95.4	99.1
Penicillin	100	97.5	96.3*	99
Clindamycin	93	94.5	90.7	91.4
Erythromycin	97.5	97	85.3	88.2
Levofloxacin	-	-	96.9	97.6
Tetracycline	21.4	8.1	9.4	11.9
Vancomycin	100	100	100	99.6
Linezolid	-	-	100	100

*p = 0.02.

### Polymicrobial and recurrent infections

*S. agalactiae* was commonly isolated with other bacteria and fungi. Invasive isolates of *S. agalactiae* were significantly involved in polymicrobial infections when compared to noninvasive isolates [13.7%, (13/95) vs 3.5% (20/576) p = 0.0002]. In 13 polymicrobial invasive infections *S. agalactiae* was concomitantly found with *Escherichia coli* (4/13), *Staphylococcus aureus* 2/13, *Klebsiella pneumoniae* 1/13, *Citrobacter freundii* 1/13, *Enterobacter aerogenes* 1/13, *Streptococcus viridans* 1/13 and *Candida albicans.* In a total of 20 noninvasive infections and colonizations *S. agalactiae* was recovered with *Escherichia coli* 4/20, *Staphylococcus aureus* 5/20 and *Candida albicans* 5/20. Polymicrobial infections were not associated with fatality (p = 0.45).

Recurrent isolation and/or colonization were recorded in the second period of study. A total of 36 (5.4%) cases with at least two documented infections or *S. agalactiae* colonizations were seen through the 2004–2012 period. One recurrent invasive infection was reported in a 67 y. o male with prostate cancer who developed osteomyelitis and epidural abscess and received a penicillin regimen. He had a relapsed two years later with positive cultures; the patient was treated with ceftriaxone.

### Microbiology of *S. agalactiae*

A total of 497 isolates were biochemically characterized. The most common biotypes comprise strains that were beta-hemolytic, resistant to bacitracin and non-carbohydrate fermenters. Non-hemolysin producing isolates emerged since 2008 and they were mostly recovered from urine samples (71% of isolates from urine vs 29% from other samples, p = 0.0001). Interestingly a small percentage of *S. agalactiae* isolates showed biochemical profiles typically seen in animal strains [21]. Four isolates (0.8%) were susceptible to bacitracin and 71 (9%) were lactose fermenters.

### Therapy

In our hospital the most common therapeutic regimen administrated in neonatal infections was the ampicillin/gentamicin combination; however, after a *S. agalactiae* positive culture, penicillin or ampicillin treatment alone was adopted. Vancomycin was mainly used to treat S*. agalactiae* infections in adults (24.5%, 13/49) followed by ceftriaxone (18.4%, 9/49) and penicillin (10.5%, 5/49). A combination therapy was administrated in many cases to cover concurrent bacterial infections or as prophylaxis to prevent them. Effective control of the underlying disease was also addressed. No bacteriological failure was documented after receiving empiric or consolidate antimicrobial regimen.

## Discussion

The estimated burden of the *S. agalactiae* disease in low- and medium-income countries remains to be determined. A recent meta-analysis estimated the global incidence of *S. agalactiae* neonatal infections to be 0.53 per 1000 livebirths with 9.6% mean case-fatality ratio [7], however few studies from low income countries were included and only one from Latin America. We have reported the incidence, epidemiology and emerging trends of *S. agalactiae* infections in our hospital over 17 years. Overall incidence of neonatal infections was 1.34 per 1000 livebirths which is 2.5 times higher than the estimated global incidence and twice higher the incidence reported for the Americas (0.67 per 1000 livebirths) [7]. Data reported from Latin América are variable and fluctuate between 0.3-1.15 × 1000 livebirths, with mortality rates ranging from 20 to 60% [5, 9, 22–27]. Neonatal incidence reported here is lower than previous reports by Bajaras et al. in Colombia [23] (neonatal incidence: 1.8 per 1000 livebirths) and similar to that reported from Brazil by Miura et al. (1.15 per 1000 livebirths 95% CI: 0.7-1.9) [24], possibly due to similar hospital characteristics. By contrast, lower mean incidence of neonatal infections has been reported by Sarubbi et al. in Argentina (0.9 per 1000 livebirths) [25], Guzmán et. al. in Chile (0.73 per 1000 livebirths) [9], Vaciloto et al. in Brazil [0.63 × 1000 livebirths (95% CI 0.13-1.85)] [26] and Solorzano in Mexico [0.65 × 1000 livebirths (95% CI 0.38- 1.11)] [27]. In this study we have seen a decline in the *S. agalactiae* neonatal incidence from 1.7 × 1000 livebirths in 1994–2001 to 1.1 per 1000 livebirths (p = 0.29) in 2004–2012. Early-onset disease remained stable whereas a substantial decreased in late-onset disease was observed. However, our data may represent even a higher incidence as it has been suggested that a real *S. agalactiae* neonatal incidence may be two or three times higher than that defined by microbiological confirmation [28].

Averaged mortality caused by neonatal infections in the 17-year period (26%) was also higher than the global case fatality ratio (9.6%) [7], nevertheless in the last 9 years a reduction in the number of fatal cases from 36.3% to 16.6% was observed, probably due to the recognition of the disease and early treatment. Similarly to previous studies, most fatalities occurred in early-onset infections and low-birth-weight infants [7].

Worldwide incidence of invasive infections in nonpregnant adults is widely variable and influenced by the population of individuals with predisposing factors. In high income countries a 2–4 fold increase in *S. agalactiae* severe infections has been recorded since 1990 [29]. Incidence of invasive infections in adults ranges from 3.4 to 7 × 100.000 in US, and from 0.3 to 0.96 × 1000 admissions in Europe [30]. In our study the behaviour and incidence of the *S. agalactiae* invasive in adults (0.75 × 1000 admissions) seems to be similar to that reported in high income countries. We observed that in 1994–2001 most of the bacteremias caused by *S. agalactiae* occurred in neonates (11/17, 64.7%), whereas in 2004–2012 these infections prevailed in adults (21.1% vs 70.1%, p = 0.002). Interestingly, *S. agalactiae* also emerged in non-infant children with predisposing conditions (0.75%). This situation is consistent with reports from other parts of the world where *S. agalactiae* infections in adults account for 75% of *S. agalactiae* invasive disease [20] and cases in non-infant children are rising [31].

Most *S. agalactiae* invasive infections and particularly *S. agalactiae* bacteremia occurred in old aged patients but in contrast with other studies [32, 33] males were the most affected. Skin and soft tissue were the main portals of entry for *S. agalactiae* blood invasion; however in most cases the source infections could not be identified. The most prevalent risk factors identified in non-pregnant adults with severe *S. agalactiae* infections were diabetes and cancer, particularly cancer of the immune system. Interestingly we reported three cases of *S. agalactiae* bone infections, formerly considered unusual, in patients with no obvious underlying conditions.

Worldwide, the overall in-hospital mortality for nonpregnant adults ranges from 3 -47%. In our study severe clinical consequences during the *S. agalactiae* infection far exceed previous reports (70% here vs 19%) [4]. A total of 19% patients with predisposing conditions and concurrent *S. agalactiae* invasive infection died. These findings are similar to those reported in Thailand, US and Taiwan with mortality rates ranging from 14% to 19% [12, 33]. As for other studies, mortality in non-pregnant adults over the two study periods had a substantial increase (0% vs 19.2%, p = 0.01) whereas neonatal mortality seems to decrease over time.

*S. agalactiae* isolates were consistently obtained from urine, vaginal swabs, blood and soft tissues. Over the study period a steady increase on the total of *S agalactiae* isolates was recorded, particularly in noninvasive isolates from urine (p = 0.02) supporting the emergence of *S. agalactiae* in urinary tract infections in our region. Similar to other studies, most urine isolates were obtained from women and >50% of the counts were < 10^5^ CFU/ml, this agrees with the suggestion that *S. agalactiae* bacteruria should be interpreted by lowering the commonly used cut off values of ≥10^5^ CFU/ml [34].

A changing trend was also seen in *S. agalactiae* invasive isolates. Soft tissue isolates declined over the last study period (p < 0.0001). A substantial shift form subcutaneous to bloodstream infections in non-pregnant adults was observed (60%, p = 0.01), this was even higher than that reported in similar studies (23-36%) [12].

Previous studies have found that important proportion of *S. agalactiae* infections are nosocomial (22%-26% [33, 35]) and polymicrobial (26%) [35]. Studies in non-pregnant adults by Skoff and Chen-Mao found 6.3 and 8.9% of polymicrobial bacteremia, *S. aureus* was the concurrent bacteria in half cases [29, 33]. Here we reported a lower overall rate of polymicrobial infections (16.8%) however the frequency of polymicrobial bacteremia in our population was higher compared with other studies. It was also seen that *Enterobacteriaceae* and other gram negative bacteria rather than *S. aureus* were co-cultured with *S agalactiae*, we assume they have been co-transferred from an endogenous source such as gastrointestinal or urinary tract. Invasive isolates were significantly more involved in polymicrobial infections.

It has been assumed that many problems regarding diagnosis and detection persist in developing countries leading to an underestimation of the *S. agalactiae* burden. In this study we also described the biochemical characteristics of *S. agalactiae* isolates in our region. The presence of non-hemolysin or low-hemolysin producing isolates may have diagnosis implications. One of the leading tests for *S. agalactiae* identification is based on its hemolytic properties and low-hemolysin producers may not be detected unless differential media are used [36]. Non-hemolysin producing isolates were frequently recovered from urine samples and blood agar was used to verify hemolysis in these isolates. Although this phenotype may reflect an intrinsic characteristic of urine isolates in this study, it is also possible that urine differential culture media may induce hemolysin inhibition; however, this will need further investigation.

*S agalactiae* phenotypes from animal origin have been previously identified in humans [37]. In a small percentage of our isolates we identified phenotypic traits described in bovine *S. agalactiae* isolates such as lactose fermentation, lack of hemolysin and susceptibility to bacitracin [21]. Furthermore, most *S. agalactiae* isolates with higher MICs for penicillin were also lactose fermenters. However any association between animal and human *S. agalactiae* isolates and resistance needs to be further elucidated.

*S. agalactiae* has been universally susceptible to penicillin and other beta lactams. One consequence of the implementation of chemoprophylaxis in industrialized countries is the increasing reports of *S. agalactiae* resistance to macrolide-lincosamide-streptogramine antibiotics and the emergence of penicillin-nonsusceptible isolates. Although in many developing countries the *S. agalactiae* prophylaxis is not widely used, macrolide and lincosamine resistance is also rising. All over the world resistance to erythromycin has been also attributed to therapy for other microorganisms [38].

In high-income countries resistance rates to erythromycin range from 3 to 54% and 1 to 43% for clindamycin [39] whereas studies in South America show resistance rates between 6 and 11% for erythromycin and around 5% for clindamycin [40, 41]. In this study *S. agalactiae* isolates examined in 2004–2012 showed a significant increase in erythromycin resistance compared to those in 1994–2001 (Figure 3) however the increase in clindamycin resistance did not reach statistically significance. These resistance rates were similar to that reported by Nakamura et. al. [41]. Here, invasive and noninvasive isolates had similar susceptibility profiles which agree with erythromycin rates reported by Garland *et al.*[39], but contrast with studies in Brazil showing higher resistance to erythromycin in colonizing isolates [42]. Although resistance mechanisms were not determined in this study, we could identified that 24.6% of the erythromycin resistant isolates were also resistant to clindamycin suggesting a MLS constitutive resistance phenotype, this is somewhat lower than other studies in South America and worldwide reporting up to 50% [39]. Resistance to tetracycline was a common feature (up to 88%) as seen elsewhere (46–91%) [38, 39, 41, 43].

Fluoroquinolone resistance may be also present in *S. agalactiae* examined in this study as we found 7 isolates with MIC >4 μg/ml for levofloxacin. Levofloxacin-resistant isolates have been reported since 2010 in Argentina [44] and Brazil [41].

So far, resistance to penicillin or ampicillin remains to be defined by the CLSI, although controversial [20], *in vitro S. agalactiae* penicillin nonsusceptibility (MIC >0.25 μg/ml) has been documented since 1995 [13, 45]. Studies by Kimura have shown that nonsusceptible *S. agalactiae* (NSGBS) are mostly recovered from nonsterile body sites, they also suggest screening for NSGBS using ceftizoxime [13]. In our study, most *S. agalactiae* isolates remained highly susceptible to penicillin and ampicillin, however for first time in our region we describe strains with presumptive reduced susceptibility to ampicillin and penicillin (MICs > 2 μg/ml). Of these, two strains exhibited MIC >8 μg/ml for penicillin which is considered extremely rare. Half isolates with MICs >2 μg/ml for penicillin showed resistance to erythromycin, which agrees with observations by Kimura [46] who found that NSGBS are significantly more resistance to erythromycin than penicillin susceptible isolates. In this work, we also found NSGBS in specimens from sterile sites (3/9), possibly for an endogenous nonsterile source.

Longtin et. al. have shown that NSGBS can be induced after long-term penicillin therapy. In the same study higher MICs to penicillin were associated with point mutations in specific penicillin binding proteins such as PBP 1a, PBP 2a and PBP 2x [45]. Although the development of resistance mechanisms in *S. agalactiae* are still under study, it is possible that susceptible adult population exposed to concomitant antibiotic penicillin or beta lactams-based therapies may be at risk of harbouring penicillin-nonsusceptible *S. agalactiae* strains.

Nevertheless, penicillin remains as the choice for the therapy of *S. agalactiae* infections. In this study vancomycin and ceftriaxone were usually administrated to cover other concurrent infections and complications in immunocompromised patients. In severe infections prolonged therapy was implemented to fully eradicate the pathogen and avoid persistence and recurrence. None of the patients reported microbiological failure suggesting a role for the immune system status and pathogen interactions in cases with clinical adverse outcome.

One limitation of this study is that only isolates from one medical centre were included, in addition NSGBS could not be confirmed by molecular methods due to the retrospective nature of the analysis. However, we think that our findings may represent the epidemiology in our region and the data may be compared with studies in similar hospitals in other countries.

## Conclusions

One of the goals for the millennium is to decrease the mortality of children under 5 years old. In our country, neonatal mortality represents 63% of the children fatalities, 43% occurring in the first week of life [47], however the impact of *S. agalactiae* on these figures is still unknown. We have shown that *S. agalactiae* infections may behave in a similar manner to those reported in high and middle income countries. This study has revealed a high incidence and lethality of early neonatal infections, emergence of severe infections in non-pregnant adults and an increase of bloodstream and urinary tract infections. All these data together with the emergence of antimicrobial resistance and tolerance, strongly support the implementation of prevention measures. We have also identified non-infant children with underlying conditions as a population at risk for invasive infections.

The epidemiology of *S. agalactiae* varies geographically and population at risk must be identified and monitored. Routinely risk-based or *S. agalactiae* swab screening in pregnant women, chemoprophylaxis and/or effective vaccine strategies should be considered in high risk population. Tertiary care hospitals in Latin America having transplantation, dialysis, cancer units or immunocompromised patients should recognize *S. agalactiae* as a severe cause of disease by monitoring and implementing policies to improve routinely detection, susceptibility testing and control. In these settings susceptibility to penicillin, clindamycin and erythromycin should be tested particularly in immunosuppressed patients at high nosocomial risk and/or those highly exposed to antibiotic therapy. Penicillin-nonsusceptible strains or isolates with a MIC range near the susceptibility breakpoint (>0.12 μg/ml) should be confirmed and further investigated to examine the potential molecular mechanism involved.

*S. agalactiae* vaccination strategies might be an effective approach to overcome *S. agalactiae* emergence and contribute to reduce the burden of *S. agalactiae* infection in the developing world. However, studies on serotype distribution, resistance mechanisms and virulence factors urge to determine its applicability in our region.

## Authors’ information

Maria del Pilar Crespo-Ortiz, MSc, PhD; Claudia Rocío Castañeda, MSc; Mónica Recalde BSc, Juan Diego Vélez, MD.Infectious diseases specialist.

## Authors’ original submitted files for images

Below are the links to the authors’ original submitted files for images. Authors’ original file for figure 1 Authors’ original file for figure 2 Authors’ original file for figure 3 Authors’ original file for figure 4

## Abbreviations

GBS: Group B Streptococcus
MIC: Minimum inhibitory concentration
CLI: Clindamycin
ERI: Erythromycin
NSGBS: Nonsusceptible GBS
PBP: Penicillin binding protein.
