# Supplementary figures and images for: Emerging trends in invasive and noninvasive isolates of Streptococcus agalactiaein a Latin American hospital: a 17-year study
Source: BMC Infect Dis. 2014 Aug 3;14:428. doi: 10.1186/1471-2334-14-428 (PMC4131052; doi:10.1186/1471-2334-14-428)

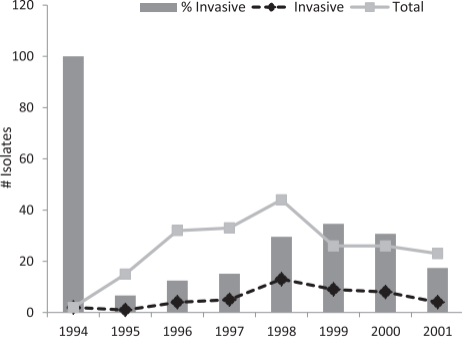

n=201

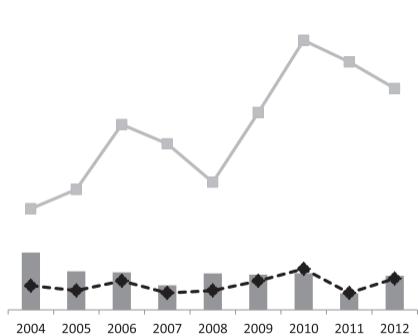

n=671

Supplement: Supplementary file 1 — Authors’ original file for figure 1 [file 12879_2014_3728_MOESM1_ESM.pdf]

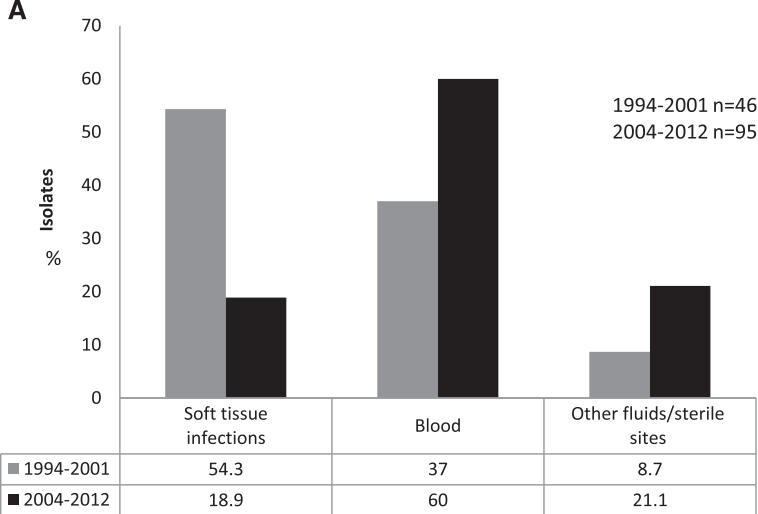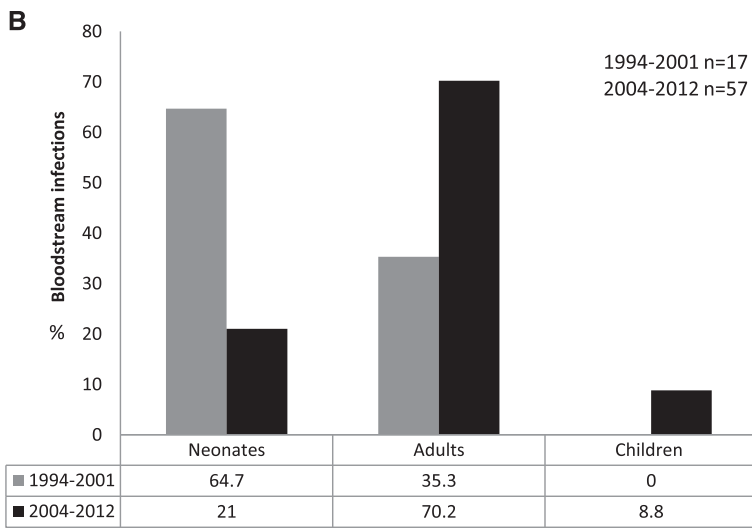

Supplement: Supplementary file 2 — Authors’ original file for figure 2 [file 12879_2014_3728_MOESM2_ESM.pdf]

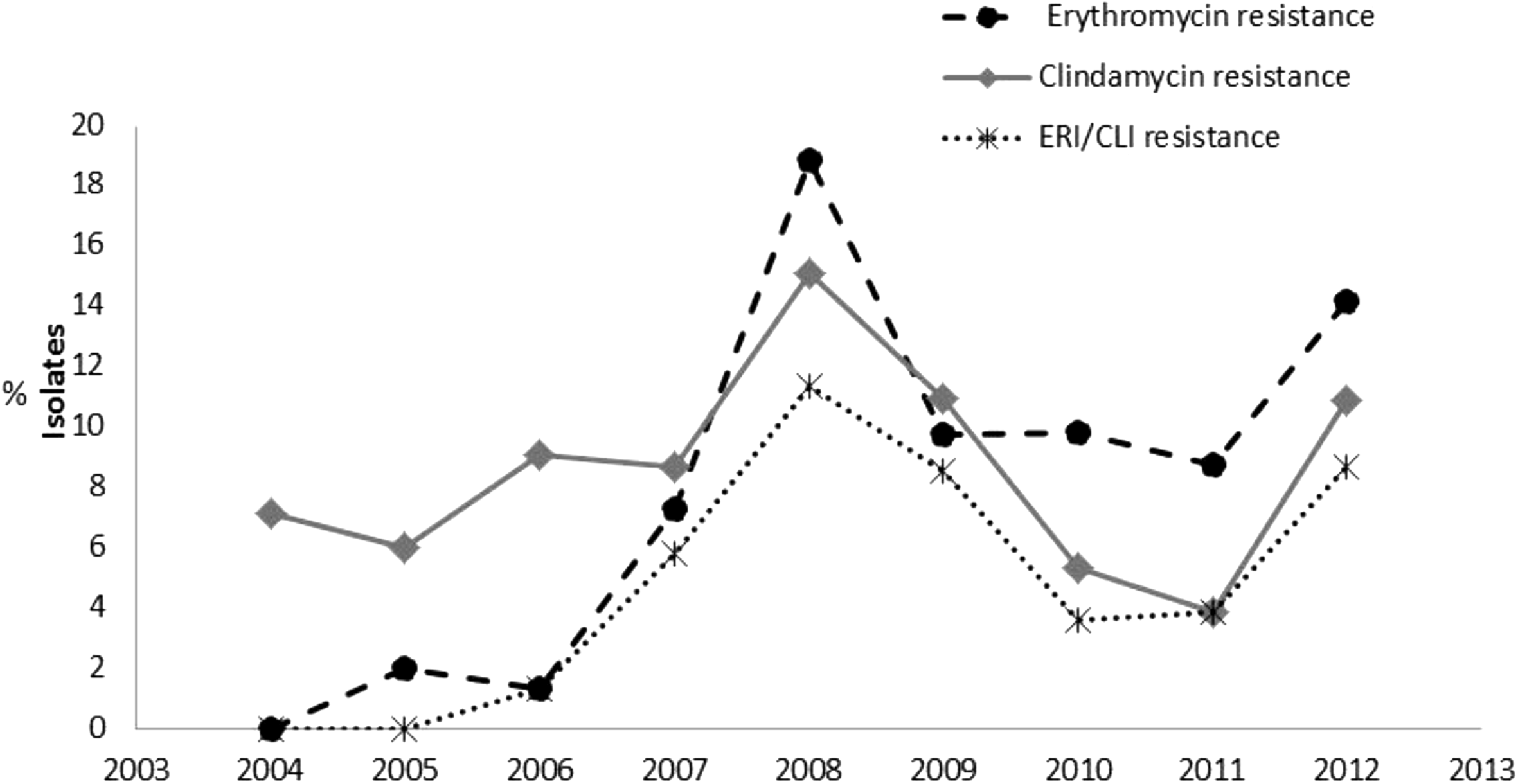

Supplement: Supplementary file 3 — Authors’ original file for figure 3 [file 12879_2014_3728_MOESM3_ESM.tif]
