# Supplementary material for: Emerging trends in invasive and noninvasive isolates of Streptococcus agalactiaein a Latin American hospital: a 17-year study
Source: BMC Infect Dis. 2014 Aug 3;14:428. doi: 10.1186/1471-2334-14-428 (PMC4131052; doi:10.1186/1471-2334-14-428)
Supplement: Supplementary file 4 — Authors’ original file for figure 4 [file 12879_2014_3728_MOESM4_ESM.docx]

Figure 4

B
